# Peanut triacylglycerols activate innate immunity both in insects and mammals

**DOI:** 10.1038/s41598-022-11494-0

**Published:** 2022-05-06

**Authors:** Wenyuan Li, Atsushi Miyashita, Kazuhisa Sekimizu

**Affiliations:** 1 Graduate School of Science and Engineering, Teikyo University, Tokyo, Japan; 2 Qinghai Provincial Key Laboratory of Traditional Chinese Medicine Research for Glycolipid Metabolic Diseases, Xining, China; 3 Institute of Medical Mycology, Teikyo University, Tokyo, Japan; 4 Graduate School of Pharma-Science, Teikyo University, Tokyo, Japan; 5 Genome Pharmaceuticals Institute, Co., Ltd, Tokyo, Japan

**Keywords:** Biochemistry, Cytokines

## Abstract

In this study, we investigated immunoreactivity of peanut (*Arachis hypogaea*) oil using the silkworm (*Bombyx mori*) model. The peanut oil induced melanin formation when injected to the silkworm hemocoel. We then purified the active substance and identified the triacylglycerols (TAGs) as the responsible molecule for the melanin-forming effect of peanut oil. Also, the peanut TAGs induced the muscle contraction of the silkworm (i.e., cleavage of the insect cytokine BmPP) and the TNF-α production by cultured mouse macrophage cells. The muscle contraction activity of the peanut TAGs was reduced by saponification reaction, indicating that the TAG (not the degraded fatty acids) moiety is responsible for the activity. The muscle contraction effects of other TAGs of olive, lard, and beef oil were comparable with that of peanut TAGs. Nevertheless, for the melanin formation, the effect of peanut TAGs was outstanding. The fatty acid composition of peanut TAGs was distinct from that of olive TAGs. These results suggest that TAGs are immunoreactive and induces cytokines both in insect and mammalian immune systems. Also, the differential effects of peanut and olive TAGs for the melanin formation may suggest that TAGs with different fatty acid compositions are distinguished by the immune system.

## Introduction

Peanut (*Arachis hypogaea* L.), a rich source of edible oil and protein, is a widely consumed food source^1^. However, peanuts are a potential health threat to humans. For example, aflatoxins (produced by the peanut mold *Aspergillus*), induces cancer, and causes liver damage^2^. Also, peanut products are a source of food poisoning by *Salmonella*^3^. Furthermore, peanut content causes a lethal shock in persons who are allergic to peanut components^4^. For the peanut allergy, our knowledge about how a person is sensitized to peanut content (i.e., underpinning molecular mechanism) is not complete. A recent study demonstrated that the lipid content of peanut induces inflammatory responses in human keratinocytes^5^, suggesting that the inflammatory responses (i.e. innate immune responses) may play a key role in the unwanted immunological reactions to peanut contents. Hence, in this study, we investigated the immunoreactivity of peanuts oil, focusing on its stimulatory effects on innate immune system, to identify the proinflammatory molecules in the peanut oil.

The major component of plant oils is triacylglycerols (TAGs). Although high blood TAGs level is associated with the risks of cardiovascular disease^6^, diabetes^7^, stroke^8^, heart disease^9^, and inflammation-related diseases^10^, direct empirical evidence for that TAGs stimulate innate immune system is relatively scarce. Some of their fatty acid moieties (TAGs are comprised of a glycerol and three fatty acid moieties), such as oleic acid, are known for immunostimulatory effects ^11^. However, the fatty acid composition differs across organismal phyla (animal TAGs tend to contain saturated fatty acids such as palmitic acid and myristic acid, while plant TAGs often contain unsaturated fatty acids such as olein acid and linoleic acid), and comparative study to understand the differential immunoreactivities of TAGs and the underpinning molecular explanation is needed.

In this study, we used the silkworm (*Bombyx mori*) as an animal model to investigate the immunoreactivity of peanut TAGs. When performing an organismal-level immunological study, invertebrate models seem useful. Invertebrates (such as the silkworm) and vertebrates shares a conserved physiology^12–18^. For example, the structure and function of Toll in invertebrates and the activation of NF-кB by Toll-like receptors (TLRs) in vertebrates are conserved^19^. Among invertebrates, we have proposed experimental models using the silkworm (*B. mori*) for understanding innate immunity^13,20–24^, infection mechanisms^25^, hyperglycemia^26^, as well as searching for beneficial natural compounds^27,28^. The pharmacokinetic parameters and toxicologic profiles of compounds observed in the silkworm model resembles those obtained in rodent model^29,30^. Furthermore, silkworm models are advantageous in that the running cost (including the bench fee for rearing facility) is small, and they are compatible with the recent growing demand for animal ethics. A trained experimenter can inject from 50 to 200 µL of liquid samples to the hemocoel of silkworm larvae, which enables quantitative toxicology/pharmacology experiments. Thus, we used the silkworm as a model animal in this study.

In this study, we started with examining the immunoreactivity of peanut oil (hexane extract) in the silkworm model. For the indexes of immunoreactivity, we used the muscle contraction assay (i.e., cleavage of insect cytokine paralytic peptide: BmPP)^20^ as well as blood melanin formation (i.e., activation of phenoloxidase (PO) cascade). We also identified the immunoreactive molecule in the peanut oil by biochemical methods. Comparative analysis using different sources of oil/fat (peanut oil, olive oil, lard, and beef fat), and different assay system (i.e., the silkworm assays in vivo and the mouse macrophage assay in vitro) is also the aim of this study.

## Results

### Peanut extract induces melanin formation in the silkworm hemolymph

**Figure 1:**
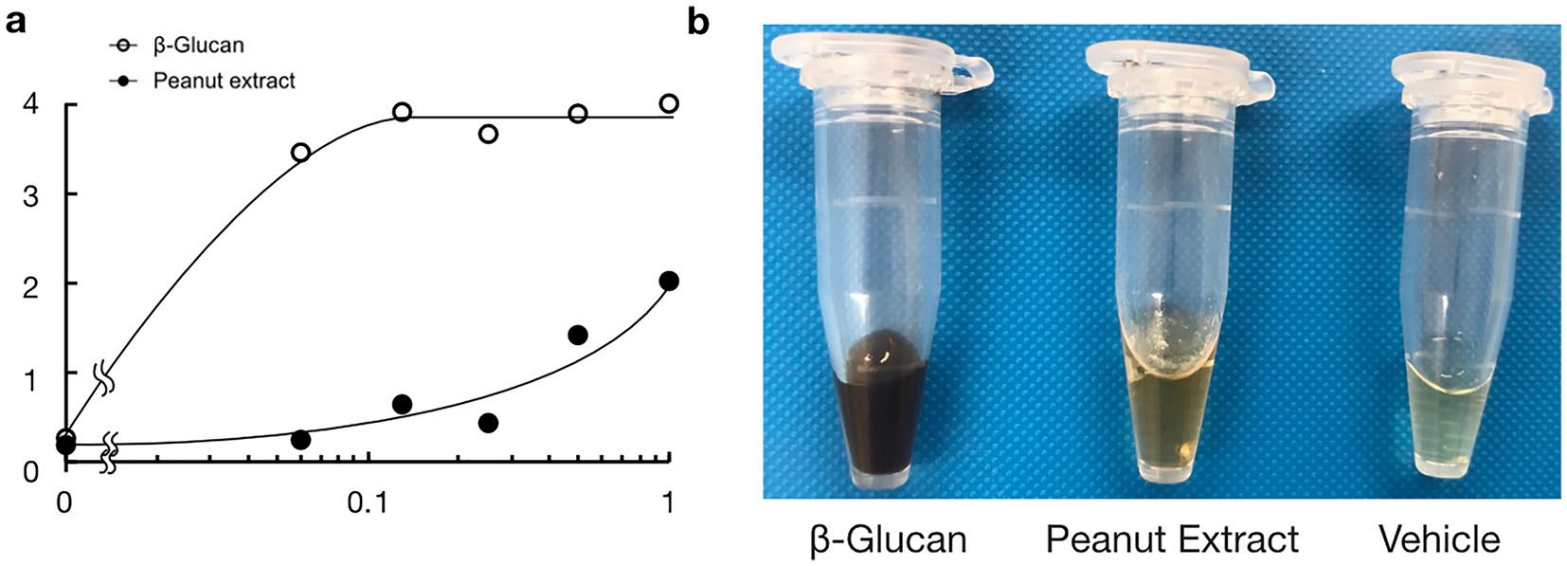
Melanization of hemolymph induced by peanut extract in silkworms. (**a**) The chart shows the dose-dependent melanin inducing effect of peanut oil in the silkworm. The y-axis represents the OD value (492 nm) of silkworm hemolymph was measured 3 h after sample injection. The x-axis represents the dose (mg/larva) of the peanut extract that was injected to the hemocoel of the silkworm. Mean values from duplicate measurements are shown in the chart. (**b**) Photo of the blackened hemolymph sample. Melanin formation was induced by injecting β-glucan (0.5 mg/larva) and the peanut extract (1 mg/larva)while the vehicle (saline) did not.

To quantify the level of melanin formation, the absorbance of hemolymph at 492 nm was measured. The absorbance of hemolymph increased in a dose-dependent manner in response to peanut extract (injected into the hemocoel) (Fig. 1a). Injection of peanut extract visibly thickened the color of hemolymph compared to the water-injected control (Fig. 1b).

### Purification/identification of the immunoreactive substance from the peanut extract

**Figure 2:**
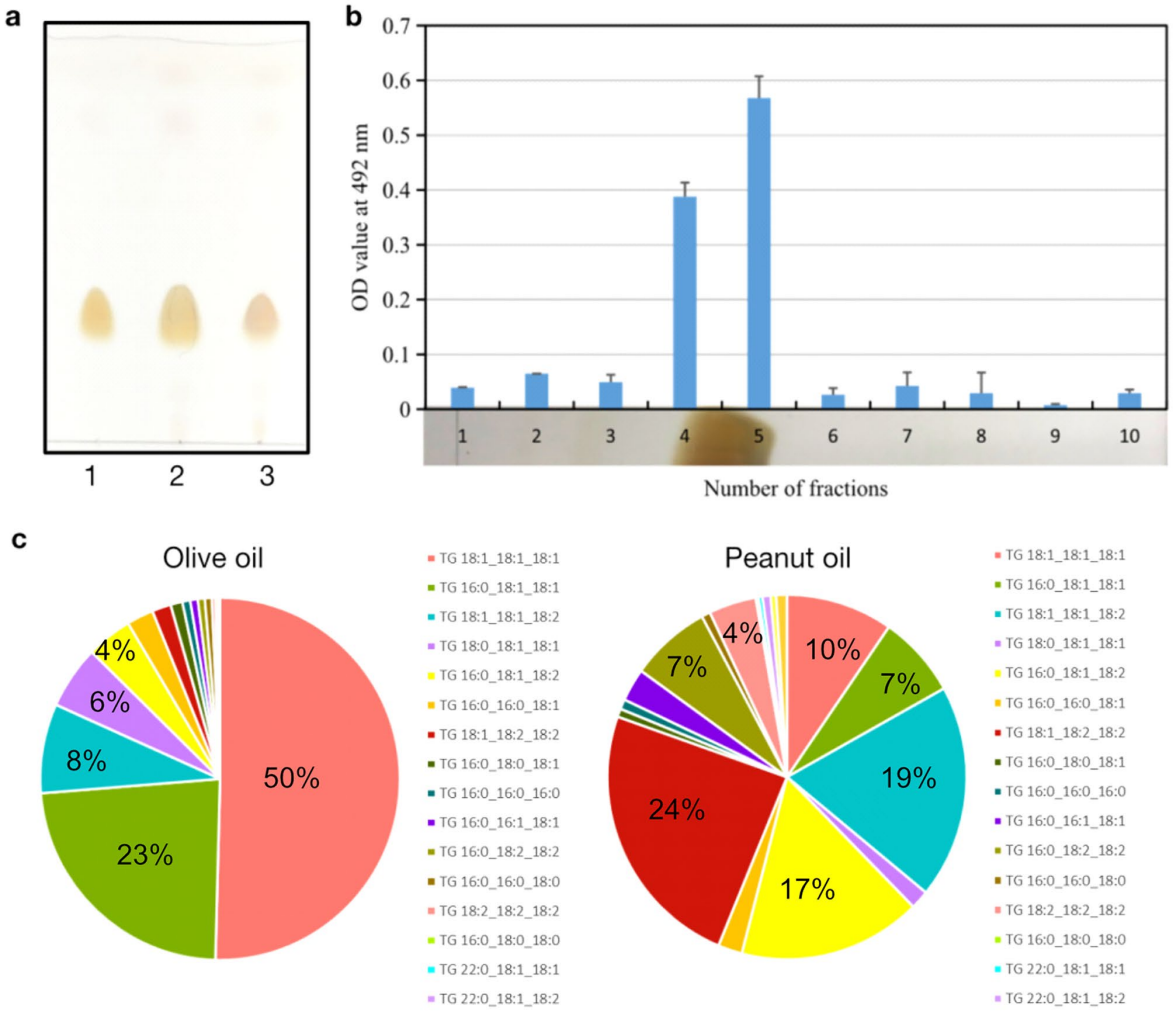
Purification of the immunoreactive substance in the peanut extract. (**a**) The immunoreactive substance was purified using a silica gel column chromatography (Ethyl acetate-Hexane 1:9 v/v), and analyzed using TLC. The samples shown in the figure are: (1) olive TAGs (control), (2) mixture of 1 and 3, and (3) the purified immunoreactive substance in the peanut extract. For the purified peanut extract, a major single spot appeared on the TLC, whose migration matched that of the olive (control) TAGs. (**b**) The immunoreactive activity (i.e., melanin formation) was recovered from the TLC spot. Each of the ten sections of silica layer as shown in the bottom panel was curved and re-extracted to examine the immunoreactivity. As shown in the chart (top panel), the immunoreactivity co-migrated with the spot. The y-axis of the top panel represents the OD value (492 nm) of silkworm hemolymph was measured 3 h after sample injection. The mean value and the standard deviation obtained from duplicate measurements are shown in the chart. (c) Fatty acid compositions were determined as described in “Materials and Methods”. Each combination of three fatty acid moieties were color-coded as demonstrated in the legend. For example, ‘TG 18:1_18:1_18:1’ represents a TAG with three oleic acid moieties, and ‘TG 16:0_18:1_18:1’ represents a TAG with a palmitic acid and two oleic acid moieties.

To purify the immunoreactive substance in the peanut extract, we loaded the peanut extract to a silica gel column and eluted using different solvents (Table 1). The specific activity (units/mg) of the fraction eluted with ethyl acetate–hexane (1:9 *v/v*) increased by twice from the loaded sample, where the yield was 93% (Table 1). Other solvents did not yield the active substance after elution (Table 1). We therefore used the Ethyl acetate-Hexane (1:9 *v/v*) eluted fraction for the following analyses. To know the chemical structure of the immunoreactive substance of the purified peanut extract (the ethyl acetate–hexane (1:9 *v/v*) fraction as shown in Table 1), we analyzed the sample using TLC. For this experiment, an olive oil sample (~ 99% TAGs) was used as a marker of triacylglycerol. The purified peanut extract showed a single spot, whose migration matched that of the olive oil sample (Fig. 2a). We further confirmed that the substance in the TLC spot shows the immunoreactivity, when re-extracted from the TLC plate, in silkworms (Fig. 2b). These results suggest that the immunoreactive substance in the peanut oil is TAGs. We then analyzed the fatty acid composition of the extracted TAG sample. As shown in Fig. 2c, the majority of olive TAGs contained 18:1/18:1/18:1 (i.e., all three fatty acid moieties were oleic acid), while that of peanut TAGs contained 18:1/18:2/18:2 (i.e., an oleic acid moiety and two linoleic acid moieties), 18:1/18:1/18:2 (i.e., two oleic acids and a linoleic acid), and 16:0/18:1/18:2 (i.e., a palmitic acid, an oleic acid, and a linoleic acid). These results are consistent with previous literature analyzing the content of the major TAGs in peanut oil^31^. It should be noted here that the analytical method used in this study does not clearly distinguish between the positions of sn-1/3 and sn-2. For example, for the TAG expressed as 18:1/18:2/18:2, this study does not distinguish whether the oleic acid (18:1) is ester bonded to the hydroxyl group at the end of the glycerol (sn-1/3) or the hydroxyl group in the center (sn-2).

**Table 1:** Silica gel column chromatography of a hexane extract of peanut.

Step	Dry mass (mg)	Total activity (units*)	Specific activity (units/mg)	Yield (%)	Purification level
Peanut extract	3000 ± 3.5	2500 ± 1800	0.84 ± 0.71	100	1
Ethyl acetate-hexane (1:9 *v/v*)	1900 ± 28	2400 ± 1000	1.25 ± 0.55	93	1.5
			**p < 0.05		

Mean values and standard errors obtained from duplicate measurements are presented in the table.

*1 unit = the dose that makes the OD_492_ value of 0.425.

**The *p* value obtained from Student’s *t*-test for the specific activities for the two fractions is shown.

### The peanut TAGs induce the insect cytokine paralytic peptide (BmPP) cleavage and lead to muscle contraction in the silkworm

**Figure 3:**
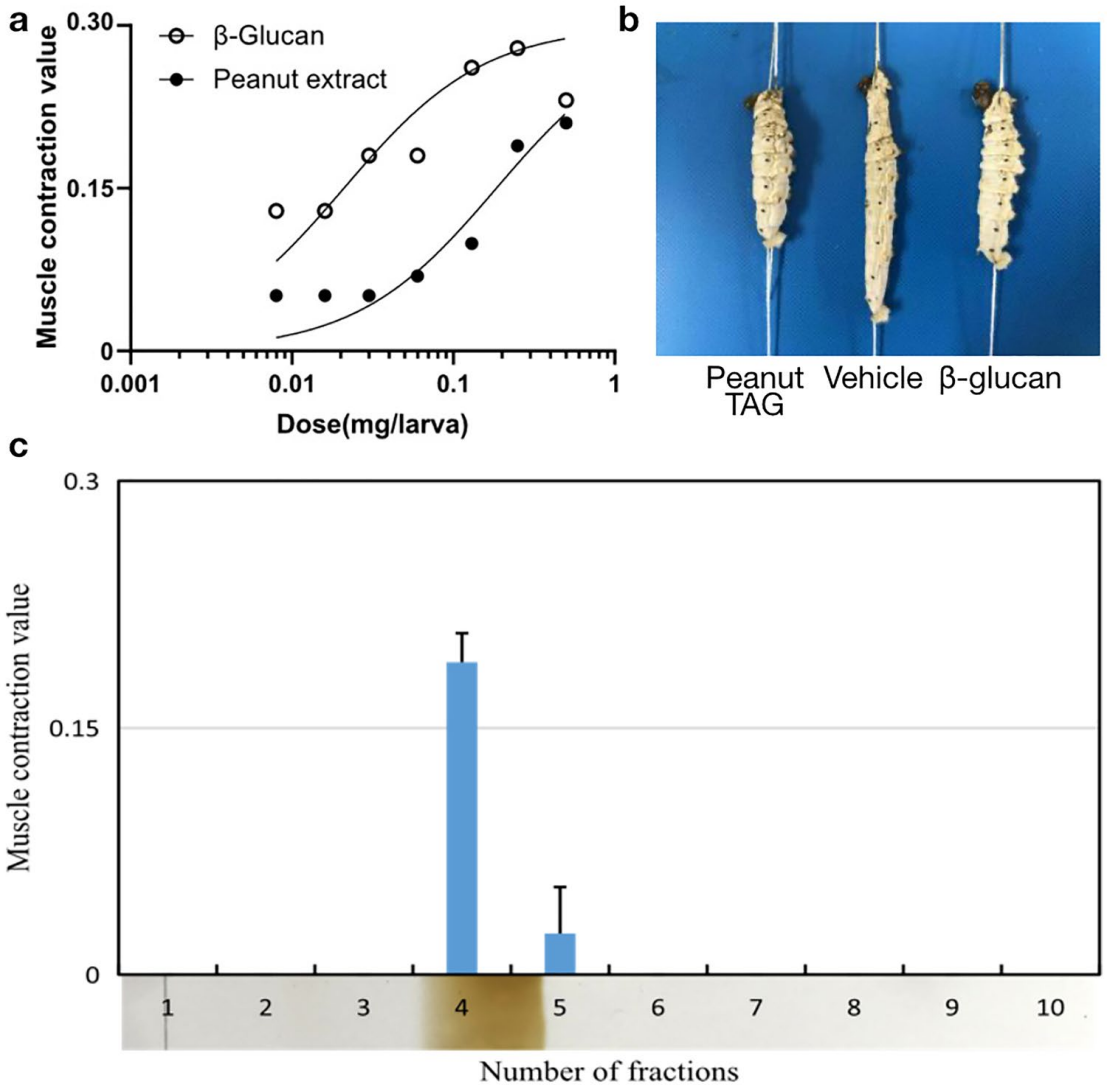
Muscle contraction of the silkworm induced by peanut TAGs. (**a**) The (purified) peanut TAGs dose-dependently showed a muscle contraction effect on the silkworm. The y-axis represents the contraction (C-) value, and the x-axis represents the dosage (mg/larva). Each plot represents a result from one silkworm specimen. Calculation procedure of C-values is described in “Materials and Methods”. (**b**) A representative photo from the muscle contraction assay using the silkworm. Either the peanut TAGs (17 mg), the vehicle (water), or the yeast β-glucan (4.0 mg) was injected to the silkworm specimen. As shown in the figure, the peanut TAG and the yeast β-glucan induced the muscle contraction, while the vehicle did not. (**c**) The muscle contraction inducing activity co-migrated with the TLC spot. Thirty milligrams of the purified peanut TAG (as shown in Fig. 2a) was separated on the TLC and re-extracted. When re-extracted, the substance in the major spot on the TLC (fraction 4 as shown in the bottom panel) induced the muscle contraction. The y-axis of the top panel represents the C-value in the muscle contraction assay. The mean value and the standard deviation obtained from duplicate measurements are shown in the chart.

We previously reported that pathogen-derived components such as beta-glucans and peptidoglycans induce activation (cleavage) of the insect cytokine paralytic peptide (BmPP), resulting in muscle contraction in the silkworm^20,32^. We examined whether the peanut TAGs induce the muscle contraction in the silkworm. As a result, the peanut TAGs induced muscle contraction, where the specific activity of the peanut TAGs was 5 units/mg and that of yeast β-glucan was 42 units/mg (Fig. 3a, b). We further confirmed that the substance in the TLC spot shows the muscle contraction activity, when re-extracted from the TLC plate (Fig. 3c). These results suggest that the triacyl glycerol may induce activation of the insect cytokine BmPP, resulting in the muscle contraction in the silkworm.

### Peanut TAGs induce cytokine production in mammalian immune system

**Figure 4:**
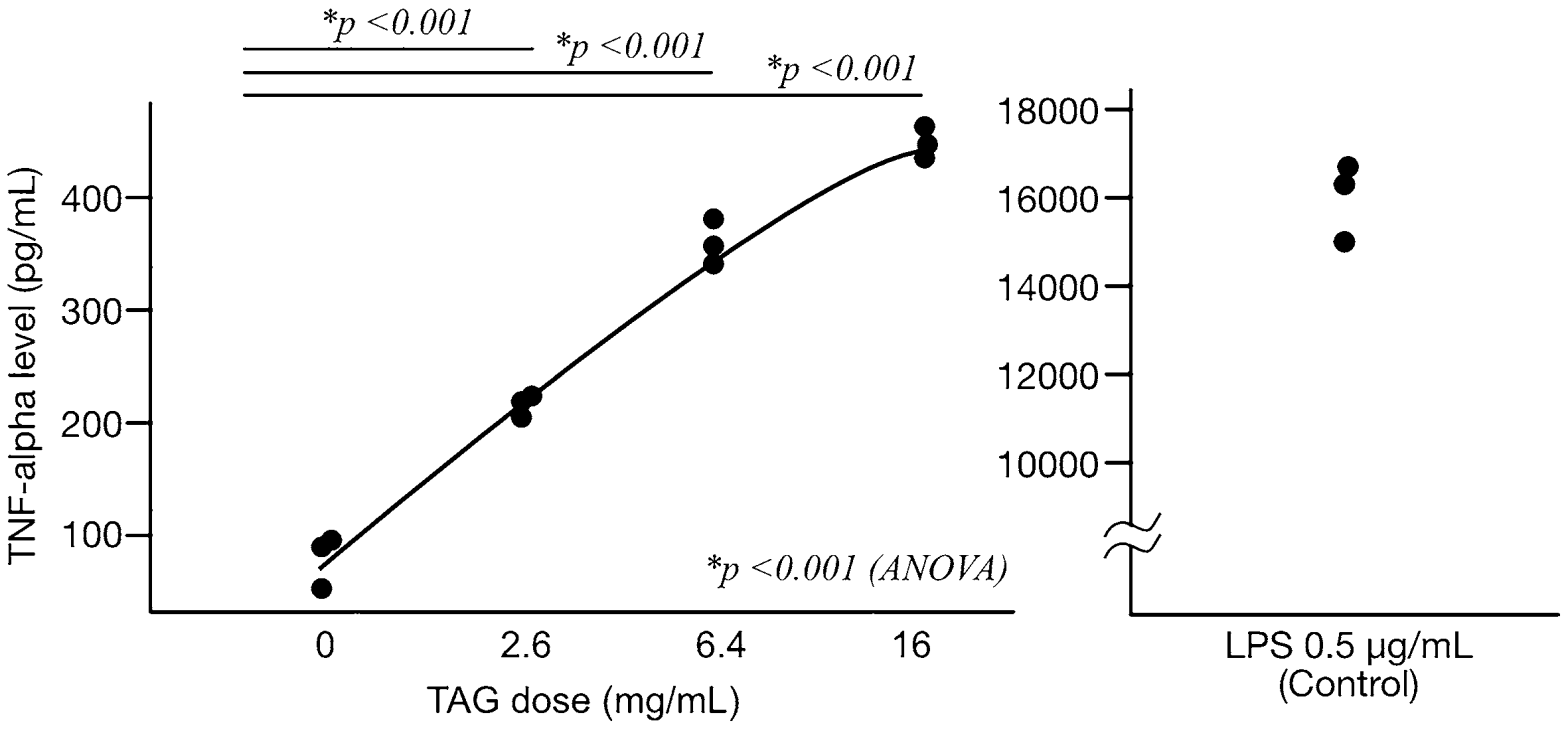
The peanut TAGs induce TNF-α production by mammalian macrophage cells. Left panel. The concentration of TNF-α present in the culture media of RAW264 cells were determined as described in “Materials and Methods”. After 6 h of co-incubation, the peanut TAGs induced TNF-α production by RAW264 cells in a dose-dependent manner. The y-axis represents the TNF-α concentration in the culture media, and the x-axis represents the dose of peanut TAGs added to the cell culture. A result of triplicate experiment is shown in the char (each plot represents one replicate). There was a significant difference across samples in the analysis of variance (ANOVA, *p* < 0.001), and each of the three doses resulted in statistically significant increases as demonstrated by the post-hoc *t*-tests (*p* < 0.001). Right panel. The concentration of TNF-α present in the culture media of RAW264 cells, when co-incubated with 0.5 μg/mL Lipopolysaccharide (LPS) for 6 h, were determined. The y-axis represents the TNF-α concentration in the culture media. A result of triplicate experiment is shown in the chart (each plot represents one replicate).

The concentrations of TNF-α found in the macrophage culture medium were 370, 280, 140, and 80 pg/mL when treated with 16, 6.4, 2.6 and 0 (vehicle control) mg/mL peanut TAGs (Fig. 4a). After a 6-h incubation with the peanut TAGs, the cells remained healthy (i.e., no death observed). These results suggest that the peanut TAGs activate cytokine cascades in mammalian immune system.

### Comparative analysis using different source of TAGs

**Figure 5:**
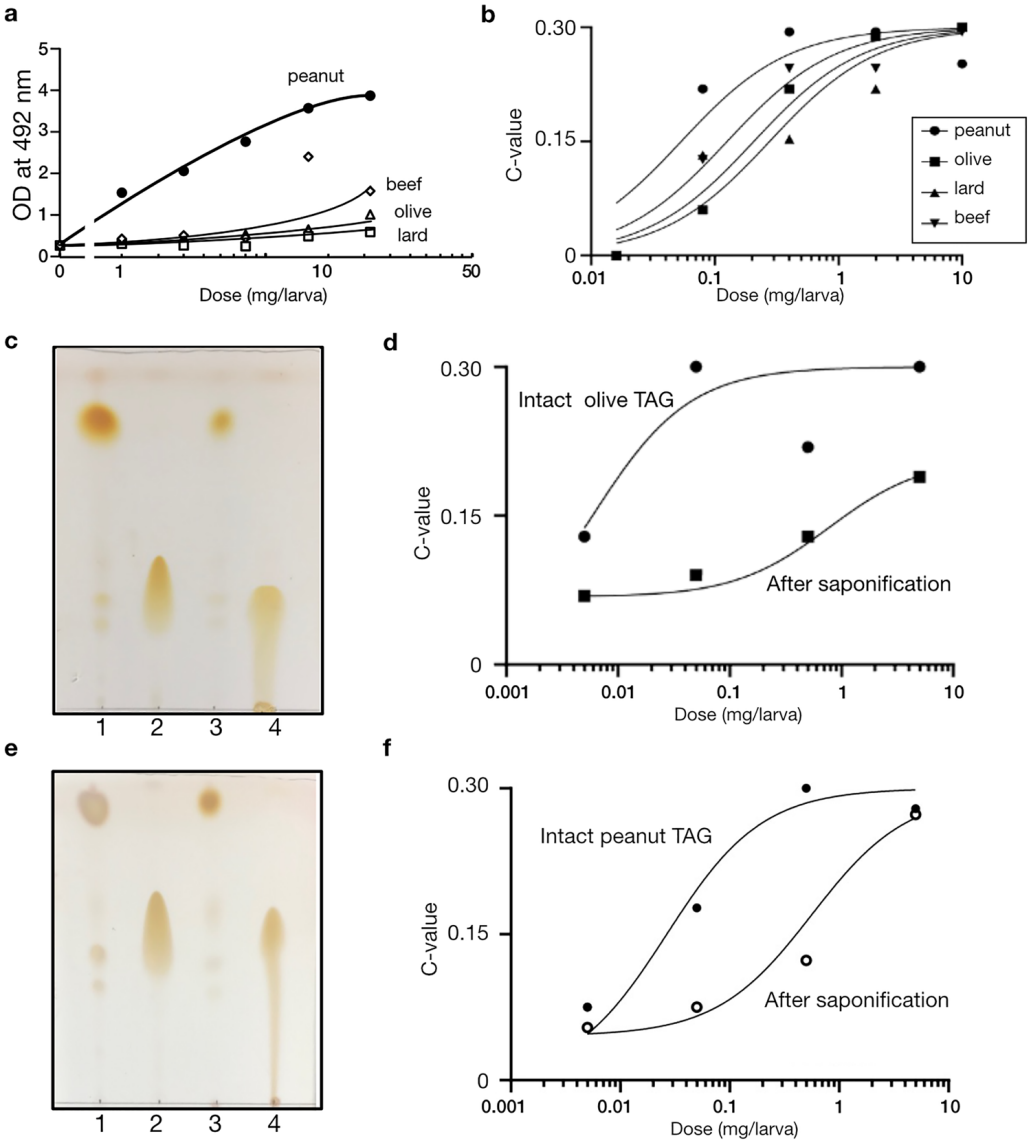
Comparative study of different sources of TAGs for the immunoreactivity. (**a**) The melanin inducing effect of the peanut TAGs was outstanding as compared to other (olive, beef fat and lard) TAGs. The y-axis of the top panel represents the OD value (492 nm) of silkworm hemolymph was measured 3 h after sample injection, and the x-axis represents the dose injected to the silkworm hemocoel (mg/larva). Mean values from duplicate measurements are shown in the chart. (**b**) The comparable muscle contraction effects of TAGs from different sources. The y-axis represents the contraction (C-) value, and the x-axis represents the dosage (mg/larva). Each plot represents a result from one silkworm specimen. (**c**) Olive TAG sample before and after the saponification reaction was analyzed by TLC. The samples shown in the panel are: (1) olive oil, (2) oleic acid, (3) olive oil mixed with NaOH without heating (i.e., before the reaction), (4) saponified olive oil. (**d**) The muscle contraction activity of olive oil was reduced by saponification reaction. The y-axis represents the contraction (C-) value, and the x-axis represents the dosage (mg/larva). Each plot represents a result from one silkworm specimen. (**e**) Peanut TAG sample before and after the saponification reaction was analyzed by TLC. The samples shown in the panel are: (1) olive oil, (2) oleic acid, (3) peanut oil mixed with NaOH without heating (i.e., before the reaction), (4) saponified peanut oil. (**f**) The muscle contraction activity of peanut oil was reduced by saponification reaction. The y-axis represents the contraction (C-) value, and the x-axis represents the dosage (mg/larva). Each plot represents a result from one silkworm specimen.

Melanin formation was compared between peanut oil (the hexane extract), beef oil, lard, and olive oil (Fig. 5a). Among them, the peanut oil showed the highest effect (specific activity was 0.5 units/mg in the presented experimental replicate). The specific activity for beef oil, lard, and olive oil was 0.1, < 0.1, < 0.1 units/mg, respectively. Also, we compared the four samples for the muscle contraction activity (i.e., cleavage of the insect cytokine BmPP), and it turned out that the peanut TAGs had the highest activity in terms of silkworm muscle contraction (Fig. 5b). Nevertheless, unlike the melanin formation, the maximum muscle contraction effect seemed comparable (i.e., when injected at an excessive dose) among the four sample, suggesting that the signaling mechanisms for the two reactions (i.e. melanin formation and muscle contraction induced by TAGs) may be independent. In this sense, the four sources of TAGs share the ability to trigger BmPP cleavage, but their affinities to the receptor may be slightly different. Also, for the muscle contraction effect, we further tested whether it is because of the TAGs per se, or because of their degradation products (e.g., fatty acids). We performed a saponification reaction for the olive oil (Fig. 5c) and peanut TAG (Fig. 5e) and found that the muscle contraction activity decreased by around 1/50 (the specific activity of olive oil = 140 units/mg, and that after saponification = 3units/mg) (Fig. 5d) and 1/20 (the specific activity of peanut oli = 130 units/mg, and that after saponification = 8 units/mg) (Fig. 5f). These results suggest that TAGs, but not fatty acids, stimulate the BmPP pathway in the silkworm.

## Discussion

In this study, we found that TAGs contained in vegetable oils and animal fats stimulate innate immunity, using the silkworm model and the confirmatory in vitro assay with mammalian macrophage. Fatty acids, the hydrolysis (saponification) products of TAGs, are known to stimulate innate immunity^33^. Analysis of the saponified products showed that it is TAGs, not fatty acids, that stimulate innate immunity in the silkworm. Furthermore, we found that TAGs promote the production of TNF-α in cultured mouse macrophage cells. This suggests that the immunoreactive effect of TAGs found in the silkworm is also common to the mammalian innate immune system. We have previously reported that silkworm hemocytes recognize pattern molecules via innate immune receptors and release reactive oxygen species, which activate (cleave) BmPP and cause muscle contraction^20^. The present results suggest that TAGs are an immunostimulatory factor and that silkworm hemocytes may recognize TAGs via innate immune receptors and release reactive oxygen species leading to cleavage of BmPP. This postulated molecular mechanism should be further validated in the future. An alternative interpretation of the present results is that sublethal damage to cells may contribute to the production of TNF-α, and the immunostimulatory effect of TAG may be indirect (e.g., by recognition of damage-associated pattern molecules (DAMPS)). This point remains unclear and needs further investigation. In invertebrates (including the silkworm), melanin formation in the hemolymph is one of the innate immune responses, where phenoloxidase (PO) is known to play an important role. In the present study, we also found that TAGs induced melanin formation (i.e., activation of PO cascade) in the silkworm. All these findings support the hypothesis that TAGs are recognized by the innate immune system and lead to inflammatory innate immune responses such as production of cytokines and effector molecules.

## Materials and methods

### Animal and cell line

Silkworms were kept as described previously^17,18,21^. Fertilized eggs of silkworm (Hu Yo × Tsukuba Ne) were purchased from Ehime Sanshu (Ehime, Japan). Hatched larvae were reared at 27 °C and fed Silkmate 2S (Nihon Nosan Kogyo, Tokyo, Japan), an artificial diet for juvenile silkworms until 4th instar. After the transition to 5th instar larval stage, they were fed Chisan 2-rei (Katakura Kogyo, Tokyo, Japan). RAW264 (RCB0535) cell line was purchased from RIKEN BRC Cell Bank (Tokyo, Japan). RAW264 cells were cultured at 37 °C and with 5% CO_2_. The animal research was conducted in compliance with all relevant guidelines and regulations applicable at the time and place of the experiments, including the ARRIVE guidelines.

### Peanut and reagents

Peanuts were purchased from Kawagoeya Co., Ltd. (Tokyo, Japan). Olive oil, lard, and beef oil were purchased at a local grocery store. Saline was purchased from Otsuka Pharmaceutical Co., Ltd. (Tokyo, Japan). Silica gel 60 F_254_ plate was purchased from EMD Millipore Corporation. Hexane, ethyl acetate, benzylpenicillin and kanamycin sulfate were purchased from Wako Fujifilm Co., Ltd. (Tokyo, Japan). Mouse TNF-α ELISA kit was purchased from R&D Systems Inc. (MN, USA). RPMI-1640 medium was purchased from Sigma-Aldrich Co., Ltd. (Buchs, Switzerland). HEPES was purchased from Dojindo Laboratories Co., Ltd. (Kumamoto, Japan).

### Preparation of peanut extract

Peanuts (50 g) were ground until a viscous state, suspended in 200 mL of hexane, and mixed rigorously. The sample was then spun at 8000 rpm for 15 min at 4 °C. The supernatant was taken and dried up using a rotary evaporator. Five microliters of samples suspended in 100 µL chloroform, 1 µL of the solution was loaded on a silica gel plate, and the plate was dipped in 30 mL ethyl acetate-hexane (1:6 *v/v*). Substances on the place was then visualized by 10% sulfuric acid.

### LC–MS/MS-based untargeted lipidomics

Lipids were extracted using single-phase extraction as described previously^34^. LC–MS/MS analysis was performed using an ACQUITY UPLC system (Waters, Milford, MA, USA) equipped with a reverse-phase column (Acquity UPLC BEH peptide C18; 2.1 × 50 mm, 1.7 μm particle size; Waters) coupled with a quadruple time-of-flight/MS (TripleTOF 6600, SCIEX, Framingham, MA, USA) as described previously^35,36^.

### Melanin formation assay using the silkworm

The 5th instar larvae (1.8–2.0 g, 2-day old after the last molt) were injected with the sample solution (50 μL) into to the hemocoel. Each sample was sonicated until homogenous state immediately before the injection. Hemolymph samples were harvested from the abdominal legs at 0, 1, 2, 3, 4, 5, and 6 h after the injection. The hemolymph samples were then spun at 10,000 rpm for 5 min at 4 ℃ to remove hemocytes and debris. The supernatant (plasma) obtained from each sample was then measured for its absorbance at 492 nm.

### Silkworm muscle contraction assay

We performed the muscle contraction assay using the silkworm as described previously^32^. The head of the silkworm was cut off using scissors, and the peritrophic membrane together with gut contents were removed. After removing the silk glands, the silkworm specimen was tied up to a transducer to measure isotonic contraction at a tension of 26 g-weight and stabilized until autonomous vibration disappeared. Sample solution (50 μL, sonicated until homogenous state before use) was injected into each specimen using a 1-mL syringe with a 27-gauge needle. The contraction value ((x – y)/x) was calculated by measuring length of each specimen before (x cm) and after (y cm) the sample injection.

### Silica gel column chromatography

Fifty grams of silica gel powder suspended in hexane was packed in a glass column. One gram of peanut extract dissolved in 10 mL of hexane was loaded to the column and washed with 200 mL hexane. Eluted fractions by 200 mL ethyl acetate-hexane (1:9 *v/v*), 200 mL ethyl acetate-hexane (1:4 *v/v*), 200 mL ethyl acetate-hexane (1:2 *v/v*), and 200 mL ethyl acetate, were collected and dried up by an evaporator and examined for their activities. Further, 10 mg of the active fraction was loaded on the silica gel plate and dipped in hexane–ethyl acetate 6:1 solvent to perform thin layer chromatography. The plate was then cut into 10 pieces and curbed the silica layer to extract substances using 30 mL ethyl acetate. Evaporated samples were then dissolved in 250 µL of water and sonicated. We confirmed that the major TLC spot contained the immunostimulatory activities.

### Quantification of melanin formation in the silkworm hemolymph induced by peanut extract

The samples from the silica gel column chromatography were diluted by 2^1^ to 2^10^ folds, and 50 µL of these samples were injected to the hemocoel of silkworms. Following an incubation at 27 °C for 4 h without feeding, we collected hemolymph from each silkworm into a plastic tube on ice (2 µL of 1 M PTU/DMSO was put in each tube in advance). Hemocytes were removed by spinning at 10,000 rpm for 5 min at 4 °C. The absorbance at 492 nm of the hemolymph was measured to quantify the levels of melanin formation. One unit of the activity was defined as the dose that correspond with the OD value of 0.425.

### Quantification of muscle contraction activity of peanut extract by using silkworms

The peanut extract (2 mg suspended in 50 µL of water, sonicated until homogenous state before use) and yeast β-glucan (2 mg suspended in 50 μL of water) were dilute by 2^1^, 2^2^, 2^3^, 2^4^, 2^5^, 2^6^, 2^7^, and 2^8^ folds. The muscle contraction value was determined for each sample as described in the preceding section. One unit was defined as the dose that induce muscle contraction with the value of 0.15.

### Measurement of TNF-α production by RAW264 cells

The RAW264 cells were cultured on 96-well plates using RPMI-1640 containing 10% fetal bovine serum (FBS). Before adding the peanut samples, culture medium was removed, and the cells were washed with RPMI-1640 (without FBS). Then, samples (and vehicle control) were added to the cell culture (100 µL/well), and the cells were incubated at 37 °C with 5% CO_2_ for 6 h. To measure the produced TNF-α, we used Mouse TNF-α ELISA kit following the manufacturer’s instruction.

### Saponification

One gram of peanut TAG sample was mixed with 800 µL of 5 N NaOH aqueous solution. The sample was analyzed by TLC before and after the reaction. For the TLC, ethyl acetate-hexane (1:4 v/v) was used as the moving solvent, and olive oil and oleic acid were used as TAG and fatty acid marker, respectively. TLC spots were visualized by 10% sulfuric acid.

### Statistical analysis

To test the differences between mean values, student's t-tests were performed using Microsoft Excel 2013. To draw the figures, we used GraphPad Prism version 8.4.3 (GraphPad Software Inc.) and Affinity Designer (Serif Europe, UK).

## Abbreviations

BmPP: *Bombyx mori* paralytic peptide
TNF: Tumor necrosis factor
TLC: Thin layer chromatography

## References

[1] Toomer OT (2018). Nutritional chemistry of the peanut (*Arachis hypogaea*). Crit. Rev. Food Sci. Nutr..

[2] Wild CP, Gong YY (2009). Mycotoxins and human disease: A largely ignored global health issue. Carcinogenesis.

[3] Scheil W, Cameron S, Heaton S, Kirk M, Vulcanis M, Holland R (1996). Human salmonellosis and peanut butter. Commun. Dis. Intell..

[4] Chang AS, Sreedharan A, Schneider KR (2013). Peanut and peanut products: A food safety perspective. Food Control.

[5] Palladino C, Breiteneder H (2018). Peanut allergens. Mol. Immunol..

[6] Harchaoui ELK, Visser EM, Kastelein JPJ, Stroes SE, Dallinga-Thie MG (2009). Triglycerides and cardiovascular risk. Curr. Cardiol. Rev..

[7] Rhee EP, Cheng S, Larson MG, Walford GA, Lewis GD, McCabe E (2011). Lipid profiling identifies a triacylglycerol signature of insulin resistance and improves diabetes prediction in humans. J. Clin. Investig..

[8] Wei YS, Lan Y, Liu YG, Meng LQ, Xu QQ, Xie HY (2009). Association of the integrin gene polymorphisms with ischemic stroke and plasma lipid levels. Zhonghua Yi Xue Yi Chuan Xue Za Zhi.

[9] Austin MA (1997). Triacylglycerol and coronary heart disease. Proc. Nutr. Soc..

[10] Zhang C, Wang K, Yang L, Liu R, Chu Y, Qin X (2018). Lipid metabolism in inflammation-related diseases. Analyst..

[11] Carrillo C, Cavia Mdel M, Alonso-Torre S (2012). Role of oleic acid in immune system; mechanism of action; A review. Nutr. Hosp..

[12] Hoffmann JA, Reichhart J-M (2002). Drosophila innate immunity: An evolutionary perspective. Nat. Immunol..

[13] Miyashita, A., & Sekimizu, K. Using silkworms to search for lactic acid bacteria that contribute to infection prevention and improvement of hyperglycemia. *Drug Discov. Therap.* 2021. 10.5582/ddt.2021.01020 33746185

[14] Miyashita A, Lee TYM, Adamo SA (2020). High-stakes decision-making by female crickets (*Gryllus texensis*): When to trade in wing muscles for eggs*. Physiol. Biochem. Zool..

[15] Miyashita, A., & Adamo, S.A. Stayin’alive: Endocrinological stress responses in insects, in *Advances in invertebrate (neuro) endocrinology* 283–323 (Apple Academic Press; 2020).

[16] Miyashita A, Lee TYM, McMillan LE, Easy R, Adamo SA (2019). Immunity for nothing and the eggs for free: Apparent lack of both physiological trade-offs and terminal reproductive investment in female crickets (*Gryllus texensis*). PLoS ONE.

[17] Miyashita A, Takahashi S, Ishii K, Sekimizu K, Kaito C (2015). Primed immune responses triggered by ingested bacteria lead to systemic infection tolerance in silkworms. PLoS ONE.

[18] Miyashita A, Kizaki H, Kawasaki K, Sekimizu K, Kaito C (2014). Primed immune responses to gram-negative peptidoglycans confer infection resistance in silkworms. J. Biol. Chem..

[19] Royet J, Dziarski R (2007). Peptidoglycan recognition proteins: Pleiotropic sensors and effectors of antimicrobial defences. Nat. Rev. Microbiol..

[20] Ishii K, Hamamoto H, Kamimura M, Nakamura Y, Noda H, Imamura K (2010). Insect cytokine paralytic peptide (PP) induces cellular and humoral immune responses in the silkworm *Bombyx mori**. J. Biol. Chem..

[21] Miyashita A, Iyoda S, Ishii K, Hamamoto H, Sekimizu K, Kaito C (2012). Lipopolysaccharide O-antigen of enterohemorrhagic *Escherichia coli* O157:H7 is required for killing both insects and mammals. FEMS Microbiol. Lett..

[22] Imae K, Saito Y, Kizaki H, Ryuno H, Mao H, Miyashita A (2016). Novel nucleoside diphosphatase contributes to *Staphylococcus aureus* virulence. J. Biol. Chem..

[23] Kaito C, Yoshikai H, Wakamatsu A, Miyashita A, Matsumoto Y, Fujiyuki T (2020). Non-pathogenic Escherichia coli acquires virulence by mutating a growth-essential LPS transporter. PLoS Pathog..

[24] Miyashita, A., Hamamoto, H., & Sekimizu ,K. Applying the silkworm model for the search of immunosuppressants. *Drug Discov Ther.* 2021. 10.5582/ddt.2021.01041 34234062

[25] Kaito C, Sekimizu K (2007). A silkworm model of pathogenic bacterial infection. Drug Discov. Ther..

[26] Matsumoto Y, Sumiya E, Sugita T, Sekimizu K (2011). An invertebrate hyperglycemic model for the identification of anti-diabetic drugs. PLoS ONE.

[27] Panthee, S., Paudel, A., Hamamoto, H., & Sekimizu, K. Advantages of the silkworm as an animal model for developing novel antimicrobial agents. *Front Microbiol*. 2017;8(373). 10.3389/fmicb.2017.00373 PMC5339274 28326075

[28] Hamamoto H, Panthee S, Paudel A, Ishii K, Yasukawa J, Su J (2021). Serum apolipoprotein A-I potentiates the therapeutic efficacy of lysocin E against *Staphylococcus aureus*. Nat. Commun..

[29] Hamamoto H, Tonoike A, Narushima K, Horie R, Sekimizu K (2009). Silkworm as a model animal to evaluate drug candidate toxicity and metabolism. Comp. Biochem. Physiol. C: Toxicol. Pharmacol..

[30] Usui K, Nishida S, Sugita T, Ueki T, Matsumoto Y, Okumura H (2016). Acute oral toxicity test of chemical compounds in silkworms. Drug Discov. Therap..

[31] Lisa M, Holcapek M (2008). Triacylglycerols profiling in plant oils important in food industry, dietetics and cosmetics using high-performance liquid chromatography-atmospheric pressure chemical ionization mass spectrometry. J. Chromatogr. A..

[32] Fujiyuki T, Hamamoto H, Ishii K, Urai M, Kataoka K, Takeda T (2012). Evaluation of innate immune stimulating activity of polysaccharides using a silkworm (*Bombyx mori*) muscle contraction assay. Drug Discov Ther..

[33] Calder PC, Grimble RF (2002). Polyunsaturated fatty acids, inflammation and immunity. Eur. J. Clin. Nutr..

[34] Tsugawa H, Ikeda K, Tanaka W, Senoo Y, Arita M, Arita M (2017). Comprehensive identification of sphingolipid species by in silico retention time and tandem mass spectral library. J. Cheminform..

[35] Naoe, S., Tsugawa, H., Takahashi, M., Ikeda, K., & Arita, M. Characterization of lipid profiles after dietary intake of polyunsaturated fatty acids using integrated untargeted and targeted lipidomics. *Metabolites.* 2019;9(10). 10.3390/metabo9100241 PMC6836067 31640217

[36] Tsugawa H, Ikeda K, Takahashi M, Satoh A, Mori Y, Uchino H (2020). A lipidome atlas in MS-DIAL 4. Nat. Biotechnol..

